# Optimization of Fermentation Conditions for Antarctic Bacteria and Investigation of Its Antimicrobial Mechanism Against *Klebsiella pneumoniae*

**DOI:** 10.3390/microorganisms13092027

**Published:** 2025-08-30

**Authors:** Lukai Xu, Mengyu Li, Yangzhu Huang, Yuanchao Mao, Shouyuan Cai, Xinyuan Yang, Xiyan Hou, Lulu Wang, Chunshan Quan, Liming Jin

**Affiliations:** Key Laboratory of Biotechnology and Bioresources Utilization of Ministry of Education, College of Life, Sciences, Dalian Minzu University, Dalian 116600, China; xvlukai@163.com (L.X.); 15825315619@163.com (M.L.); h534198987@163.com (Y.H.); maoyc2023@126.com (Y.M.); 18265502153@163.com (S.C.); syyy-@outlook.com (X.Y.); xyhous@dlnu.edu.cn (X.H.); wanglulu0813@126.com (L.W.)

**Keywords:** *Klebsiella pneumoniae*, Antarctic bacteria, *Bacillus nakamurai*, bacteriostatic mechanism

## Abstract

*Klebsiella pneumoniae* is the second-most common opportunistic pathogen in clinical practice and has developed resistance to potent antibacterial drugs such as carbapenems. Therefore, developing safe and effective strategies for the prevention and treatment of *K. pneumoniae* infections remains a critical challenge. In this study, a strain named Tie-10 isolated from Antarctic samples demonstrated potent antibacterial activity against *K. pneumoniae*, which was subsequently identified as *Bacillus nakamurai*. The fermentation medium and culture conditions were systematically optimized through single-factor experiments, orthogonal array testing, and response surface methodology. The optimal medium composition was determined to be beef extract, peptone, and KNO_3_. The culture conditions included a time of 24 h, temperature of 37 °C, pH of 7.0, and bottling volume of 80 mL. Antagonistic experiments demonstrated that the crude extract of *B. nakamurai* Tie-10 exhibited significant inhibitory activity against *K. pneumoniae*. The alkaline protease (AKP) assay demonstrated that the crude extract effectively disrupted the cellular integrity of *K. pneumoniae*, a finding further corroborated by scanning electron microscopy (SEM) analysis. Furthermore, the crude extract significantly inhibited extracellular protease secretion in *K. pneumoniae*, downregulated the expression of virulence-associated genes, and effectively disrupted biofilm formation. The study presented innovative strategies for the management and containment of *K. pneumoniae* infections.

## 1. Introduction

*Klebsiella pneumoniae* is a prevalent Gram-negative pathogen commonly found in the human respiratory and gastrointestinal tracts [1]. As an opportunistic pathogen, it primarily causes infections in individuals with compromised immune systems. *K. pneumoniae* is responsible for a range of severe infections, including pneumonia, sepsis, meningitis, and liver abscesses, posing a significant threat, particularly to immunocompromised patients. The virulence of *K. pneumoniae* is largely attributed to its diverse array of virulence factors, such as capsular polysaccharides, lipopolysaccharides, adhesins, and siderophores [2]. These factors facilitate bacterial colonization within the host and enable the pathogen to evade immune system clearance [3]. In recent years, the overuse of antibiotics has exacerbated the issue of antibiotic resistance in *K. pneumoniae*, particularly its resistance to multiple drugs. *K. pneumoniae* develops resistance to conventional antibiotics through mechanisms such as the production of β-lactamases and the loss of outer membrane porins, which complicate treatment efforts. In hospital environments, *K. pneumoniae* can spread rapidly through contact, especially in intensive care units (ICUs) [4]. Consequently, developing novel strategies for the prevention and control of *K. pneumoniae* infections represents a critical research priority.

Antarctica, the most extremely cold place on Earth, presents a unique combination of environmental stressors, including hyper-aridity, prolonged freezing temperatures, and high-intensity UV radiation [5]. These severe conditions have been selected for microbial communities with extraordinary adaptive capacities, which are reflected in their production of structurally novel bioactive metabolites with potent antimicrobial properties [6,7]. Recent bioprospecting efforts have identified antibacterial compounds from psychrophilic bacteria, filamentous fungi, and microalgae indigenous to Antarctic ecosystems, showing remarkable efficacy against clinically relevant pathogens (methicillin-resistant *Staphylococcus aureus* and extended-spectrum β-lactamase-producing *Escherichia coli*) [8]. Nevertheless, our current understanding represents merely a fraction of Antarctica microbial biosynthetic potential, with more than 90% of taxa remaining uncultured and uncharacterized [9]. Key knowledge gaps persist regarding molecular targets and resistance mechanisms, optimization of fermentation protocols for polar strains, and translational applications in medicine and agriculture. Systematic exploration of these cryophilic microorganisms may yield breakthrough solutions for addressing the global antimicrobial resistance crisis through discovery of next-generation antibiotics and eco-friendly biocontrol agents.

Given this context, Antarctic microorganisms are hypothesized to synthesize unique antimicrobial compounds that differ structurally and functionally from conventional antibiotics, presenting significant potential for the discovery and development of innovative therapeutic agents against *K. pneumoniae*. As a multidrug-resistant pathogen, *K. pneumoniae* poses urgent challenges in clinical settings, necessitating the exploration of unconventional ecological niches for novel antibacterial solutions. The extreme conditions of Antarctica have driven the evolution of highly specialized microbes with adaptive metabolic capabilities, making them promising sources of compounds active against resistant pathogens like *K. pneumoniae*. This study aimed to isolate a bacterium from Antarctica and evaluate the inhibitory activity and mechanism of action of *Bacillus nakamurai* Tie-10 against *K. pneumoniae*. We believe that this research will provide a theoretical basis for inhibiting *K. pneumoniae*.

## 2. Materials and Methods

### 2.1. Antarctic Sample, Bacterial Strain, and Culture Medium

The Antarctic samples comprised seal feces, penguin feces, and soils from the vicinity of Lake Kitek and Lake Tuanjie. These samples were taken by the 2016–2017 Antarctic Scientific Expedition and were preserved at the laboratory 605, School of Life Sciences, Dalian Minzu University.

Experimental strains were purchased from the BeNa Culture Collection Center (Shanghai, China).

Basic fermentation medium: yeast extract (5 g), tryptone (10 g), NaCl (10 g), and distilled water (up to 1 L).

### 2.2. Isolation and Screening of Antarctic Microorganisms

The isolation of Antarctic microorganisms was conducted using standard serial dilution techniques. Initially, a 10^−1^ stock suspension was prepared by homogenizing 1 g of Antarctic environmental samples in 9 mL of sterile distilled water. Serial dilutions ranging from 10^−6^ to 10^−8^ were subsequently prepared from this stock [10]. Aliquots (50 μL) from each dilution were aseptically spread-plated onto seven distinct culture media: nutrient agar (NA), Luria–Bertani agar (LA), brain heart infusion agar (BHI), De Man–Rogosa–Sharpe agar (MRS), R2A agar, Czapek–Dox agar, and potato dextrose agar (PDA). To maximize microbial recovery, plates were incubated under three temperature regimes (12 °C, 28 °C, and 37 °C). Pure cultures were obtained through successive streak-plating on fresh media. Colony morphology assessment served as the primary criterion for initial microbial identification. Representative isolates exhibiting distinct colonial characteristics were cryopreserved in 50% (*v*/*v*) glycerol solution at −80 °C for long-term storage.

The primary screening for evaluating the antimicrobial potential of the isolated microorganisms was performed using the face-off plate method. Briefly, the *K. pneumoniae* suspension (1 × 10^8^ CFU/mL) was inoculated into NA medium at 55 °C and allowed to solidify. The pure isolates were then streaked onto the NA plate in a straight line. The plates were incubated at 37 °C for 12 h, after which the presence of inhibition zones was observed. Microbial strains exhibiting significant activity were selected for secondary screening, which was conducted using the agar well method with fermented broth against the test pathogens. Briefly, the selected Antarctic bacteria were inoculated into LB medium and cultured in liquid for 24 h. The culture was then concentrated using a rotary evaporator. Wells 12 mm in diameter were created in the agar plates, and 300 μL of the fermented broth was added to each well [11]. The plates were incubated at 37 °C for 24 h. After incubation, the inhibition zones were measured to identify antibacterial compounds produced by the potential isolate. All experiments were performed in triplicate, and the average values were used for analysis.

### 2.3. Identification of Antagonistic Microbial Strains

The best strains obtained from the screening were streaked onto LA medium to obtain pure cultures. The morphology, color, and other characteristics of single colonies were observed and recorded. The 16S rDNA sequence was amplified using the universal bacterial primers 27F (5′-AGAGTTTGATCMTGGCTCAG-3′) and 1492R (5′-TACGGYTACCTTGTTACGACTT-3′). The cycling parameters were as follows: (1) initial denaturation at 94 °C for 5 min; (2) 30 cycles of denaturation at 94 °C for 30 s, annealing at 55 °C for 30 s, and extension at 72 °C for 2 min; and (3) final extension at 72 °C for 10 min [12,13]. The PCR products were sent to Sangon Biotech Co., Ltd. (Shanghai, China) for sequencing. The obtained sequences were submitted to the NCBI database (accession number: PX210274) to determine the putative identity of the strain, and a polygene phylogenetic tree was constructed using MEGA 11.0.

### 2.4. Inhibition Spectrum of the Antagonistic Bacteria

This study evaluated the inhibition spectrum of selected antagonistic strains against ten clinically relevant pathogens, including *K. pneumoniae* and other common opportunistic bacteria (Table 1). The standardized agar diffusion assay is detailed in Section 2.2.

### 2.5. Screening for Optimal Ingredients in Fermentation Medium

The optimal culture medium was screened using a modified version of Sa’s method [14], with systematic variations in fermentation media composition. LB medium served as the control, supplemented with additional components, including beef extract, calcium carbonate, sucrose, fructose, and glucose. Multiple nitrogen sources were evaluated for their anti-*K. pneumoniae* activity, including yeast extract, peptone, urea, ammonium sulfate, and potassium nitrate. NaCl was sequentially replaced with MgSO_4_, KNO_3_, KH_2_PO_4_, and CaCl_2_, and the antimicrobial efficacy against *K. pneumoniae* was assessed in each experimental group. Control groups were established by individually omitting a carbon source, a nitrogen source, or inorganic salts. Subsequently, the effects of varying concentrations of each factor on the bacteriostatic activity were examined, following the methodology described in Section 2.2.

### 2.6. Single-Factor of Fermentation Parameters on the Antibacterial Activity of Tie-10

Following the determination of the optimal culture medium composition, a single-factor test was conducted to evaluate the impact on varying fermentation conditions to the inhibitory activity of Tie-10 [15,16]. The baseline conditions were set as follows: bottling volume of 100 mL, fermentation time of 24 h, pH of 7.0, and temperature of 37 °C. To assess the effect of individual factors, only one condition was altered at a time while keeping the others constant. The tested conditions included bottling volumes: 60 mL, 80 mL, 100 mL, 120 mL, 140 mL, and 160 mL; pH: 4, 5, 6, 7, 8, 9, and 10; temperatures: 17 °C, 22 °C, 27 °C, 32 °C, 37 °C, 42 °C, and 47 °C; and times: 12 h, 24 h, 36 h, 48 h, 60 h, 72 h, and 84 h. The antibacterial activity was evaluated following the experimental procedures outlined in Section 2.2.

### 2.7. Response Surface Optimization Test

Based on the results of the single-factor tests, the four key parameters—bottling volume (A), pH (B), temperature (C), and time (D)—were selected for optimization. Response surface optimization was designed using the Box–Behnken method, implemented with Design-Expert 13 software [17], as detailed in Table 2.

### 2.8. Preparation and Characterization of Antibacterial Crude Extracts from Tie-10

The Tie-10 strain was subjected to large-scale cultivation under optimized nutritional and environmental parameters. The harvested fermentation broth underwent sequential solvent extraction, first with ethyl acetate, followed by methanol, yielding a crude extract. Antimicrobial efficacy was quantitatively determined via well-diffusion bioassays. Further investigations were then conducted to delineate the underlying antibacterial mechanisms of the prepared extract.

### 2.9. Determination of Minimum Inhibitory Concentration (MIC) of Crude Extracts Against K. pneumoniae

The minimum inhibitory concentration (MIC) of the crude extract against *K. pneumoniae* was determined using the broth microdilution method [18]. Briefly, 100 μL of NB medium was added to each well of a 96-well plate, followed by the addition of 100 μL of crude extract (100 mg/mL) to the first well. Serial two-fold dilutions were performed to generate a concentration gradient ranging from 50 mg/mL to 0.012 mg/mL. Subsequently, 100 μL of an activated *K. pneumoniae* suspension (1 × 10^8^ CFU/mL) was inoculated into wells containing different concentrations of the crude extract, with 12 experimental groups and three replicates per group. Controls included a positive control (*K. pneumoniae* inoculated in NB medium without crude extract) and a negative control (medium containing different concentrations of crude extract without *K. pneumoniae*). The plate was incubated at 37 °C for 24 h in a constant temperature incubator. The MIC was defined as the lowest concentration of the crude extract at which the well remained clear after incubation and upon gentle agitation.

### 2.10. Effects of the Crude Extract on the Growth Curve of K.pneumoniae

The antibacterial activity of the crude extract on the *K. pneumoniae* growth curve was assessed according to established protocols [19]. *K. pneumoniae* suspension was introduced into NB medium at an inoculation density of 0.1% (*v*/*v*). Test groups received varying concentrations of the extract (ranging from 1/8 to 1 MIC), while control groups contained sterile NB medium alone. Bacterial growth was monitored spectrophotometrically through hourly sampling, with optical density measurements recorded at a 600 nm wavelength. Growth inhibition profiles were generated by plotting temporal changes in absorbance values against incubation time.

### 2.11. Viability Assessment of K. pneumoniae by PI/DAPI Fluorescent Staining

*K. pneumoniae* was cultured until achieving mid-log phase growth before treatment with the crude extract at MIC. An untreated *K. pneumoniae* suspension served as the negative control in all experimental assays [20]. Following 4 h of co-incubation, cells were harvested by centrifugation (4000× *g*, 10 min, 4 °C) and washed twice with sterile physiological saline (0.85% NaCl). Viability staining was performed sequentially using (1) propidium iodide (PI) to identify membrane-compromised cells and (2) 4′,6-diamidino-2-phenylindole (DAPI) for total cell enumeration, according to the manufacturer’s specifications. Stained preparations were immobilized on microscope slides and immediately visualized using epifluorescence microscopy (400× magnification). Cell viability ratios were calculated from triplicate counts of PI-positive (non-viable) vs. DAPI-positive (total) populations.

### 2.12. Effects of Crude Extract on AKP Content in Culture Medium

The crude extract was added to NB medium at concentrations of 1/2 MIC, 1 MIC, and 2 MIC, respectively, followed by inoculation with *K. pneumoniae*. Each concentration was tested in triplicate. In the control group, *K. pneumoniae* was inoculated into NB medium containing an equivalent volume of fermentation medium. Cultures were incubated in a shaker at 37 °C and 180 rpm/min, with aliquots collected at 2 h intervals over an 8 h period. Subsequently, the samples were centrifuged at 7000 rpm for 10 min at 4 °C to collect the bacterial cells. Alkaline phosphatase (AKP) activity was measured using the AKP Assay Kit (Jiancheng Bioengineering Institute, Nanjing, Jiangsu, China) at a wavelength of 520 nm [21].

### 2.13. Effects of Crude Extract on Cell Morphology of K. pneumoniae

Scanning electron microscopy (SEM) was used to observe changes in cell morphology [22,23]. Briefly, *K. pneumoniae* was incubated in NB broth, and the crude extract was added to achieve a final concentration of 1 MIC. A sample without the crude extract served as the control group. The cultures were incubated in a shaker at 180 rpm and 37 °C for 4 h. The bacterial suspension was centrifuged at 8000× *g* for 10 min at 4 °C to harvest the bacterial cells. Subsequently, the cells were washed three times with PBS and fixed with 2.5% glutaraldehyde at 4 °C overnight. Following three PBS washes (5 min each), cellular dehydration was achieved through sequential ethanol treatments (25%, 50%, 75%, and 100% *v*/*v*) with 10-min incubations at each concentration. The samples were then deposited onto silicon wafers. Following conductive coating, the morphology of *K. pneumoniae* cells was examined using SEM (S-4800, Hitachi, Tokyo, Japan). The electron gun acceleration voltage was 5 kV, and the resolutions were 5 µm, 3 µm, 2 µm, and 1 µm, respectively, and images were collected.

### 2.14. Effects of Crude Extracts on Extracellular Protease Secretion in K. pneumoniae

The inhibitory effects of crude extract on extracellular protease secretion by *K. pneumoniae* were evaluated using a modified milk plate assay [24]. Sterile skim milk (2% *w/v* in PBS) was aseptically incorporated into NA medium at a 1:1 (*v*/*v*) ratio to prepare casein-containing medium. Test plates were supplemented with crude extract at sub-inhibitory concentrations (1/8, 1/4, 1/2, and 1 MIC). An untreated plate containing only the culture medium was established as the negative control. The well (12 mm diameter) was aseptically punched into the plate and inoculated with 300 μL of mid-log phase *K. pneumoniae* suspension. Following 48-h incubation at 37 °C, proteolytic activity was quantified by measuring hydrolysis zone diameters (including colony growth) using digital calipers with 0.01 mm resolution. Three independent experimental replicates were performed.

### 2.15. Effects of Crude Extract on Biofilm Formation in K. pneumoniae

The ability of the crude extract to inhibit *K. pneumoniae* biofilm formation was evaluated using a semi-quantitative crystal violet staining method [25]. *K. pneumoniae* was inoculated into a 24-well plate, and the crude extract was added to achieve final concentrations of 1/2 MIC, 1 MIC, and 2 MIC, with three replicates for each concentration. In the control group, *K. pneumoniae* was inoculated into an equal volume of NB medium in a separate 24-well plate. The plates were incubated at 37 °C for 48 h, followed by rinsing with 0.01 mol/L PBS and fixating with 1 mL methanol. Subsequently, 500 μL of 0.1% crystal violet solution was added to all wells containing completely dried biofilms. After 30 min of dark staining, excess crystal violet was removed by washing three times with PBS. The plates were inverted and dried in a 37 °C oven before adding 500 μL of 33% glacial acetic acid. The plates were then placed on a shaker for 30 min to dissolve the crystal violet. Finally, 50 μL of the resulting solution was transferred to a new 96-well plate, and the absorbance was measured at 590 nm. The biofilm inhibition rate was calculated using Equation (1) [26]:

Inhibition rate (%) = (OD positive control − OD assay)/OD positive control × 100%(1)

### 2.16. Effects of Crude Extracts on Virulence Gene Expression in K. pneumoniae

Mid-log phase bacterial cultures were treated with crude extract at 1 MIC final concentration, while parallel control groups received equivalent volumes of sterile solvent. Following 4 h incubation under agitation, bacterial cells were pelleted by centrifugation (12,000× *g*, 10 min, 4 °C). Total RNA was isolated from *K. pneumoniae* using the UNIQ-10 Trizol Total RNA Extraction Kit (Sangon Biotech (Shanghai) Co., Ltd., Shanghai, China) according to the manufacturer’s protocol. RNA integrity was verified by agarose gel electrophoresis, and cDNA synthesis was performed using reverse transcriptase. Gene expression analysis targeted three key virulence determinants: *luxS*, *wabG*, and *fimH*. Quantitative real-time PCR (qRT-PCR) was conducted in triplicate using the primer sets detailed in Table 3 [27], with 16S rRNA serving as the endogenous control.

### 2.17. Statistical Analysis

All experiments were performed in triplicate, and results were expressed as mean ± standard error (SE). SPSS 22.0 (IBM, Armonk, NY, USA) and Microsoft Excel 2021 (Microsoft Corporation, Redmond, WA, USA) were used for statistical analysis and data processing. One-way analysis of variance (ANOVA) followed by the least significant difference (LSD) post hoc test were applied to assess the effects of different medium components and fermentation conditions on the antibacterial activity of Tie-10. Statistical significance was set at *p* < 0.05. Data visualization was conducted using GraphPad Prism 9.5 (GraphPad Software, San Diego, CA, USA).

## 3. Results

### 3.1. Screening and Identification of Antagonistic Strains

**Figure 1:**
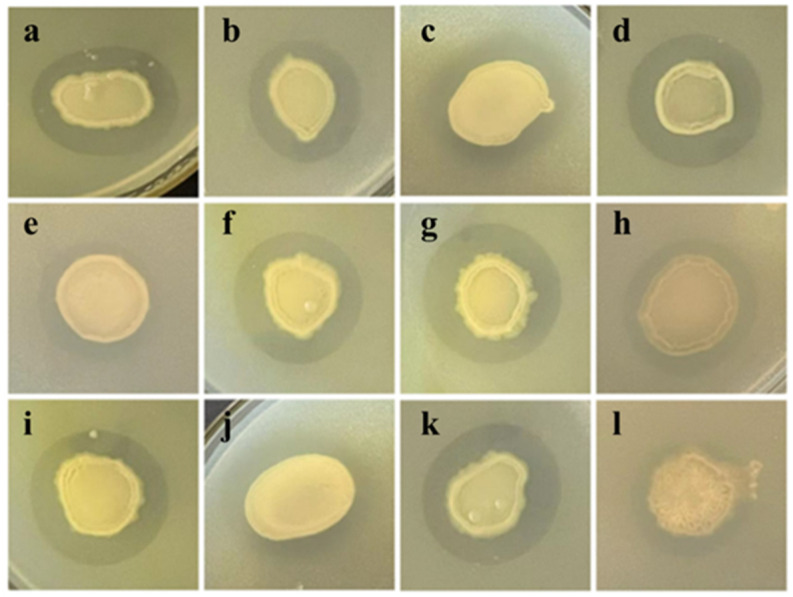
Result of preliminary screening. Tie-41 (**a**), Tie-34 (**b**), HB-12 (**c**), TJ-10 (**d**), qe-45 (**e**), TJ-40 (**f**), Tie-10 (**g**), C-7 (**h**), TJ-59 (**i**), HB-4 (**j**), TJ-3 (**k**), and qe-35 (**l**).

Initial isolation from Antarctic samples yielded 289 bacterial strains, among which 52 demonstrated inhibitory activity against *K. pneumoniae* in primary screening assays (Figure 1). Secondary screening identified Tie-10 as exhibiting the most potent antimicrobial activity, forming a distinct inhibition zone of 25.22 ± 0.27 mm diameter in standardized disk diffusion assays (Table 4).

**Figure 2:**
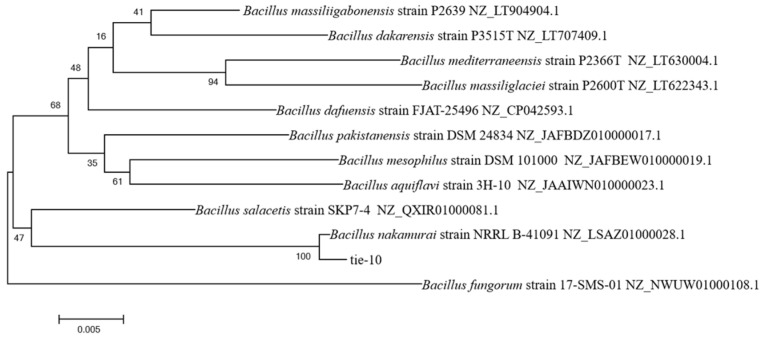
Phylogenetic tree of *B. nakamurai* Tie-10 based on 16S rDNA gene sequences, constructed using the maximum likelihood method with MEGA 11.0 software. Bootstrap values are based on 1000.

Consequently, Tie-10 was selected for further investigation. Sequence comparison using BLAST (https://blast.ncbi.nlm.nih.gov/Blast.cgi) on NCBI revealed significant homology between Tie-10 and *Bacillus nakamurai* at the nucleic acid level. The similarity of the 16S rDNA sequence was 99.58% (query cover: 99%). Furthermore, phylogenetic analysis using MEGA 11 to construct a polygene phylogenetic tree indicated that Tie-10 and *B. nakamurai* clustered together on the same branch with a bootstrap value of 100% (Figure 2).

### 3.2. Inhibition Spectrum of Tie-10

**Figure 3:**
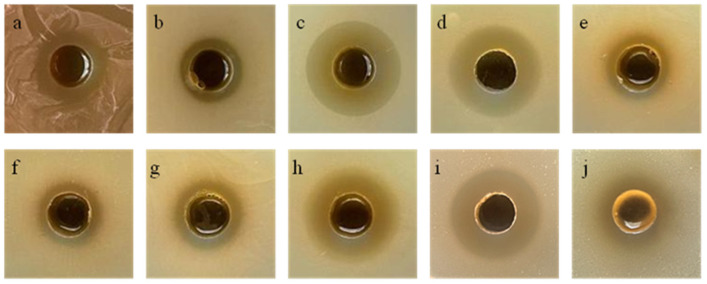
Inhibition spectrum of Tie-10. *Enterococcus faecium* (**a**), *Acinetobacter baumannii* (**b**), *Klebsiella pneumoniae* (**c**), *Escherichia coli* (**d**), *Staphylococcus aureus* (**e**), *Pseudomonas aeruginosa* (**f**), *Vibrio parahaemolyticus* (**g**), *Salmonella typhimurium* (**h**), ESBL *E. coli* (**i**), and MRSA (**j**).

The metabolites derived from Tie-10 exhibited inhibitory activity against ten common pathogenic bacteria (Table 5). Notably, Tie-10 demonstrated potent inhibitory effects on both *K. pneumoniae* and *E. coli*, with inhibition zone diameters of 25.41 ± 0.44 mm and 25.25 ± 0.12 mm, respectively (Figure 3).

### 3.3. Results of Fermentation Medium Components Through Systematic Screening

**Figure 4:**
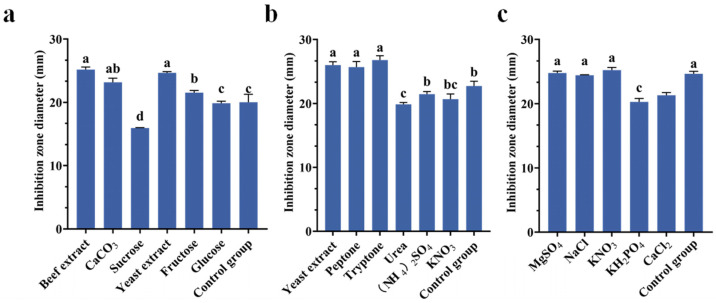
Effect of different carbon sources (**a**), nitrogen source (**b**), and inorganic salt (**c**) on the inhibition zone diameter of Tie-10. Vertical bars represent the standard error, and different letters indicate significant differences between groups (*n* = 3, *p* < 0.05).

The growth performance of Tie-10 varied across different culture media, resulting in differences in the antibacterial activities of its metabolite crude extracts against *K. pneumoniae*. The results demonstrated that variations in medium composition significantly affected the inhibition zone diameter of the Tie-10 fermentation solution. Notably, the fermentation medium containing beef extract (Figure 4a), pancreatic peptone (Figure 4b), and KNO_3_ (Figure 4c) yielded significantly larger inhibition zones against *K. pneumoniae* compared to other formulations (*p* < 0.05). Strain Tie-10 exhibited maximal antimicrobial activity with zone diameters of 25.19 ± 0.40 mm, 26.79 ± 0.69 mm, and 25.23 ± 0.42 mm.

### 3.4. Results of Single-Factor Experimental Analysis

**Figure 5:**
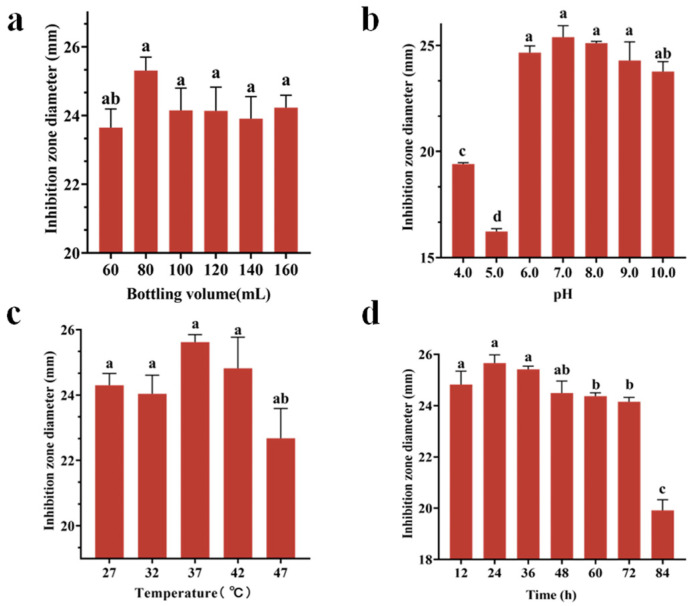
Effect of bottling volume (**a**), pH (**b**), temperature (**c**), and time (**d**) on the inhibition zone diameter of Tie-10. Vertical bars represent the standard error, and different letters indicate significant differences between groups (*n* = 3, *p* < 0.05).

The antibacterial efficacy of Tie-10 fermentation products demonstrated significant dependence on the cultivation parameters, exhibiting optimal bioactivity under specific fermentation conditions. Maximum inhibition zone diameters were achieved at an 80 mL working volume (25.31 ± 0.39 mm, *p* < 0.05) (Figure 5a), pH 7.0 (25.39 ± 0.54 mm, *p* < 0.05) (Figure 5b), 37 °C incubation temperature (25.62 ± 0.23 mm, *p* < 0.05) (Figure 5c), and 24-h fermentation duration (25.66 ± 0.32 mm, *p* < 0.05) (Figure 5d). The antimicrobial activity of the fermentation products displayed characteristic parabolic responses to each parameter, with performance declining at both suboptimal and supraoptimal conditions. These findings collectively demonstrated the critical importance of precise parameter control for maximizing the bioactive potential of Tie-10.

### 3.5. Optimization Outcomes from Response Surface Methodology Experiments

The design and results of the response surface test are presented in Table S1.

#### 3.5.1. Regression Equation and Analysis of Variance

A regression equation model was established through statistical analysis of the experimental data (Table S1) to predict the inhibition zone diameter (mm), as described by the following equation: = 27.94 + 0.4275A − 0.4508B−0.2483C + 0.145D + 0.23AB + 0.5725AC + 0.895AD + 0.415BC−0.3825BD − 0.5275CD − 1.4A^2^ − 1.31B^2^ − 1.4C^2^ − 2.43D^2^.

Variance analysis and significance testing were performed on the regression model, with the results presented in Table S2. The regression model was statistically significant (*p* < 0.0001). The primary terms A and B were significant (*p* < 0.05), whereas the primary terms C and D were not significant. Additionally, the squared terms A^2^, B^2^, C^2^, and D^2^ exhibited considerable antifungal activity. The ecoefficiency of determination (R^2^ = 0.9329) indicated a strong correlation within the model. The F-value reflected the relative importance of each factor on the inhibition zone diameter, with higher F-values corresponding to greater influences. The analysis revealed that the order of influence on the inhibition zone diameter was as follows: pH (B) > bottling volume (A, mL) > temperature (C, °C) > time (D, h).

#### 3.5.2. Response Surface Analysis of the Interaction of Various Factors

**Figure 6:**
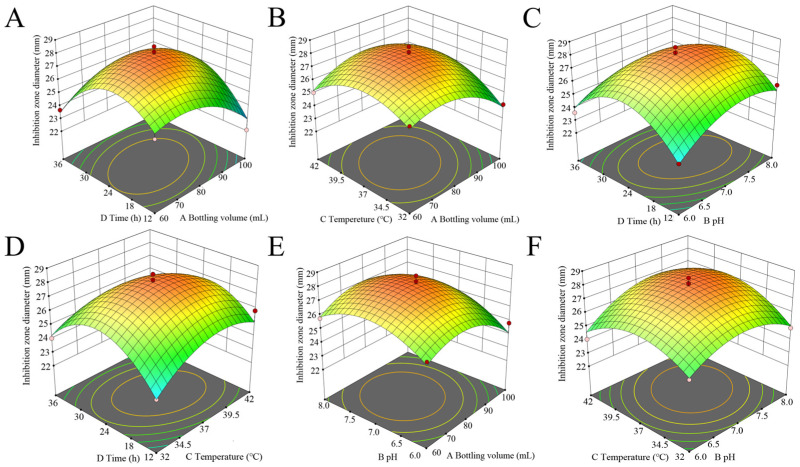
The response surface methodology and contour plots of the effects of the interaction between bottling volume and time (**A**), bottling volume and temperature (**B**), pH and time (**C**), temperature and time (**D**), bottling volume and pH (**E**), and pH and temperature (**F**) on the inhibition zone diameter of the Tie-10 crude extract. Note: The color gradient in the figure represents the variation in response values, with red denoting high values, yellow intermediate values, and green low values.

The response surface diagram presented in Figure 6 provided an intuitive representation of the interactions among the four factors and their effects on the inhibition zone diameter. The slope of the surface diagram directly correlated with the influence of the factors on the response value. A larger focal length of the contour indicated a stronger interaction between parameters. Figure 6 demonstrated that the interaction between variables A (bottling volume) and D (time) significantly affected the antibacterial efficacy of Tie-10. Using a quadratic polynomial regression fitting equation, the optimal conditions were determined as follows: bottling volume—80.657 mL, pH—6.814, temperature—36.632 °C, and time—24.056 h. Under these conditions, the predicted inhibition zone diameter is estimated to be 28.038 mm. Therefore, the optimal fermentation conditions were a bottling volume of 80 mL, a pH of 7.0, a temperature of 37 °C, and a time of 24 h.

### 3.6. MIC Determination of the Crude Extract

As the concentration of the crude extract decreased, the optical density at 600 nm (OD_600_) increased, indicating an elevation in the turbidity of the culture medium in the 96-well plate. At a crude extract concentration of 1.56 mg/mL, the culture in the well plate appeared clear and exhibited an 80% inhibition rate against *K. pneumoniae* growth (Table 6). Therefore, the MIC of the crude extract against *K. pneumoniae* was determined to be 1.56 mg/mL.

### 3.7. Effect of the Crude Extract on Growth Curve of K. pneumoniae

**Figure 7:**
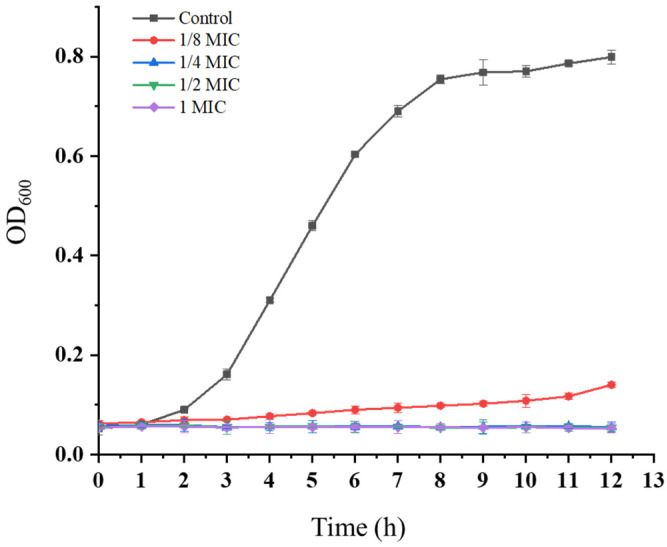
Dynamic changes in the *K. pneumoniae* growth curve. Vertical bars represent the standard error (*n* = 3).

The growth curve of *K. pneumoniae* in response to the crude extract was evaluated by monitoring the OD_600_ over time. In the control group, the bacteria exhibited typical growth dynamics, entering the logarithmic phase within 2 h and reaching the stationary phase by 8 h (Figure 7). In contrast, treatment with the crude extract at 1 MIC, 1/2 MIC, and 1/4 MIC resulted in significant growth suppression, with OD_600_ values remaining below 0.1 throughout the experiment. At the subinhibitory concentration of 1/8 MIC, *K. pneumoniae* showed delayed growth, exceeding an OD_600_ of 0.1 only after 9 h of incubation.

These findings demonstrated a clear concentration-dependent inhibitory effect; as higher extract concentrations progressed, bacterial growth was reduced across all growth phases. The growth curve analysis confirmed that the crude extract significantly impeded *K. pneumoniae* proliferation, with near-complete inhibition observed at 1/4 MIC.

### 3.8. Antimicrobial Efficacy of the Crude Extracts Against K. pneumoniae

**Figure 8:**
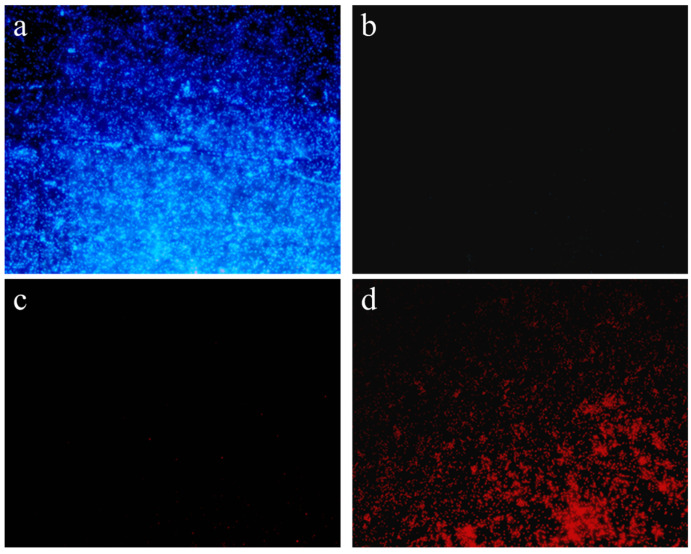
Assessment of *K. pneumoniae* viability. Control group (**a**,**c**) and treatment group (**b**,**d**).

In the untreated control group, *K. pneumoniae* exhibited a low mortality rate, as evidenced by only sporadic red fluorescent spots. This observation suggested that, under standard culture conditions, *K. pneumoniae* cells at this growth stage maintained a high viability. In contrast, treatment with the crude extract significantly increased bacterial cell death, as demonstrated in Figure 8d. The treated group displayed a marked rise in red fluorescent signals compared to the control, indicating widespread loss of membrane integrity and a higher proportion of non-viable cells.

### 3.9. Results of AKP Content in Culture Supernatants

**Figure 9:**
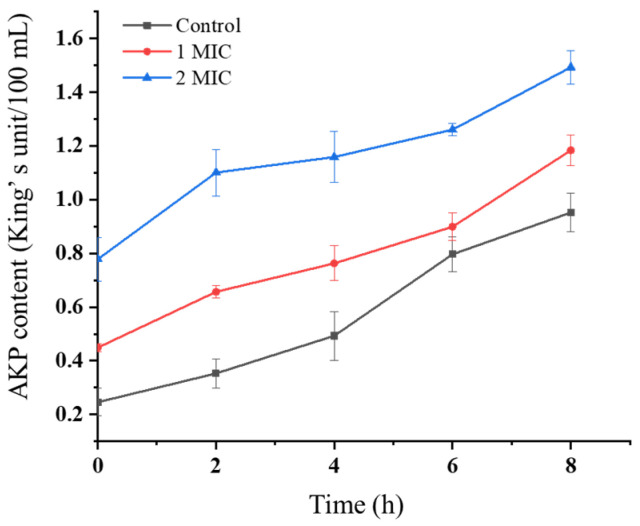
AKP content by *K. pneumoniae* across treatment groups. Vertical bars represent the standard error (*n* = 3).

The experimental results indicated a positive correlation between incubation duration and AKP accumulation in the culture medium (Figure 9). Concurrently, elevated crude extract concentrations consistently resulted in higher extracellular AKP levels at each measured timepoint. These findings supported the hypothesis that the crude extract induced cellular structural damage in *K. pneumoniae*, facilitating AKP leakage from the periplasmic space.

### 3.10. Morphological Characterization of K. pneumoniae Cells

**Figure 10:**
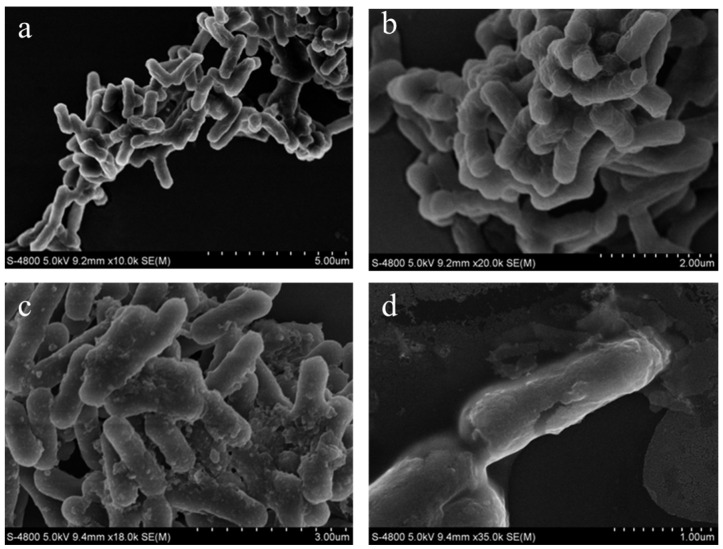
Sample of the morphology of *K. pneumoniae*. Control group (**a**) and treatment group (**b**–**d**).

SEM observations confirmed the effects of the crude extract (1 MIC) on *K. pneumoniae* cells (Figure 10). Control group cells maintained structural integrity, displaying characteristic rod-shaped morphology with uniform dimensions and intact cell membranes. In contrast, crude extract treatment induced severe membrane disruption, evidenced by cellular content leakage and loss of structural organization. These morphological alterations confirmed the bactericidal activity of the extract by directly compromising the cellular structural integrity.

### 3.11. Inhibitory Effects of Crude Extracts on Extracellular Protease Secretion in K. pneumoniae

**Figure 11:**
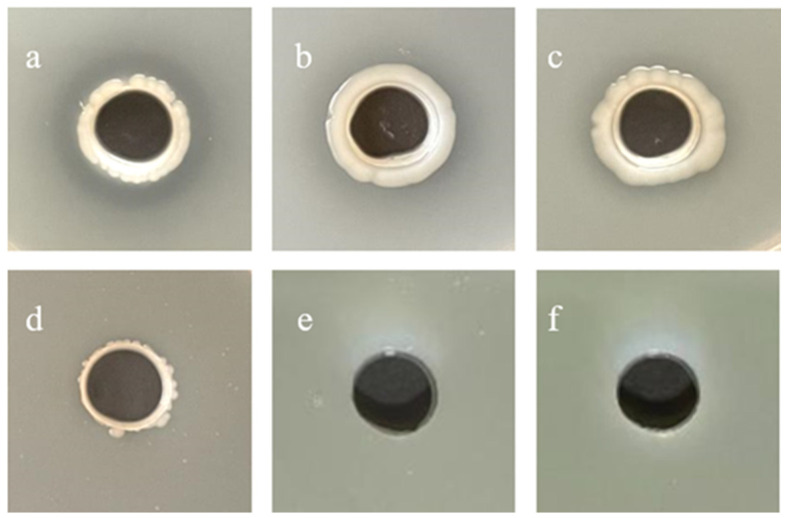
Determination plate of extracellular protease. Negative control (**a**), 1/8 MIC (**b**), 1/4 MIC (**c**), 1/2 MIC (**d**), 1 MIC (**e**), and 2 MIC (**f**).

Control experiments demonstrated that untreated *K. pneumoniae* actively secreted extracellular proteases, evidenced by clear hydrolytic zones in milk agar plates due to casein degradation. Quantitative analysis revealed a concentration-dependent inhibition of proteolytic activity by the crude extract. At 1/4 MIC, a significant reduction in zone diameter was observed. Notably, 1/2 MIC treatment completely abolished the proteolytic capability while permitting limited bacterial growth, whereas the 1 MIC resulted in complete growth suppression (Figure 11). These findings suggested a dual antimicrobial mechanism: primary inhibition of extracellular protease secretion followed by secondary growth arrest.

### 3.12. Inhibitory Effects of the Crude Extract on Biofilm Formation in K. pneumoniae

The crude extract exhibited concentration-dependent inhibition of *K. pneumoniae* biofilm formation (Table 7). Significant biofilm suppression was observed at 1/2 MIC, with the maximum inhibition reaching 51.63% at 2 MIC treatment. This dose–response relationship demonstrated the potent anti-biofilm activity of the extract, which nearly completely inhibited biofilm development at supra-inhibitory concentrations.

### 3.13. Evaluation of the Crude Extract on Gene Expression Levels in K. pneumoniae

**Figure 12:**
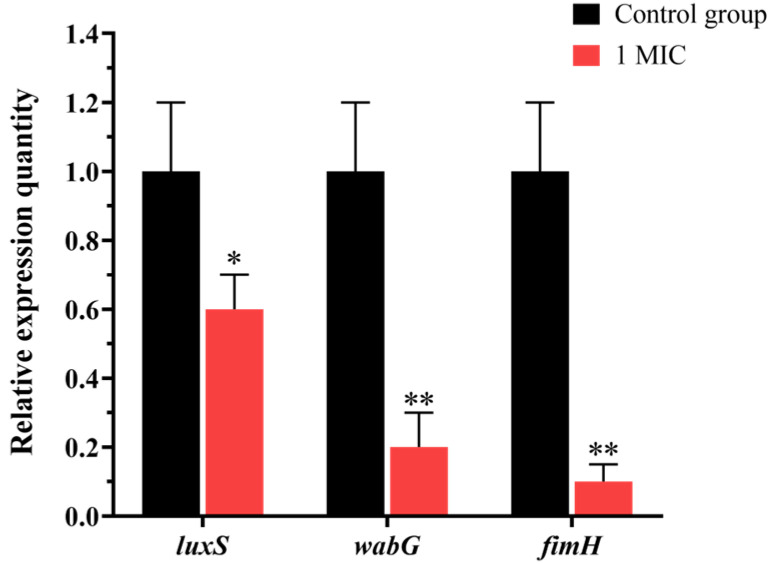
Effect of sample on gene expression of *luxS*, *wabG*, and *fimH*. Vertical bars represent the standard error, and different numbers of asterisks indicate significant differences between groups (*n* = 3, *p* < 0.05).

The quantitative analysis of gene expression levels in *K. pneumoniae* demonstrated significant downregulation of *luxS*, *wabG*, and *fimH* following treatment with the fermentation crude extract. As shown in Figure 10, the relative expression levels of these genes were markedly reduced to 0.6-fold, 0.19-fold, and 0.1-fold compared to the control group (Figure 12). These results indicated that the crude extract effectively suppressed the expression of key virulence-associated genes: *luxS*, *wabG*, and *fimH*. The substantial reduction in expression levels, particularly for *wabG* and *fimH*, suggested potent inhibitory effects on bacterial pathogenicity mechanisms.

## 4. Discussion

The escalating virulence and expanding antibiotic resistance profiles of *K. pneumoniae* represent a persistent and growing threat to global health security. Combating this multidrug-resistant pathogen necessitates a coordinated, multidisciplinary approach integrating innovative biomedical research, evidence-based clinical interventions, and comprehensive public health initiatives [28]. Antarctic microbiota has evolved distinctive biochemical pathways in response to environmental stressors, resulting in the production of structurally unique antimicrobial agents. Empirical studies validate their efficacy against resistant pathogens. The fungal strain *Aspergillus flavus* HDN151418, isolated from an Antarctic marine sponge, produced secondary metabolites demonstrating potent broad-spectrum antimicrobial activity. Bioassay results revealed significant growth inhibition against *Bacillus cereus*, MRSA, and *Vibrio parahaemolyticus* bacterial pathogens [29]. Despite mounting evidence demonstrating the antimicrobial potential of Antarctic microbiota, current research efforts remained inadequate to fully exploit their biotechnological applications. While numerous studies have confirmed the efficacy of polar microbial metabolites against pathogenic bacteria, significant gaps persist in strain characterization, compound optimization, and translational development. This investigation successfully isolated several Antarctic bacterial strains exhibiting anti-*K. pneumoniae* activity, with strain Tie-10 demonstrating the most potent inhibition. Molecular identification via 16S rDNA sequencing classified Tie-10 as *B. nakamurai*, a species displaying broad-spectrum antimicrobial properties against multiple clinically relevant pathogens.

Optimal culture medium composition and fermentation parameters are critical determinants of microbial metabolite production. This study identified the crude extract of Tie-10 as exhibiting maximal anti-*K. pneumoniae* activity. Through systematic optimization, the ideal medium formulation was established as containing beef extract, pancreatic peptone, and potassium nitrate in aqueous solution. Among the evaluated fermentation parameters, pH exerted the most pronounced effect on antibacterial production, followed sequentially by culture volume (80 mL/250 mL flask), incubation temperature (37 °C), and duration (24 h). The optimized conditions yielded a 28.11 ± 0.35 mm inhibition zone against *K. pneumoniae*, improved by 2.89 mm compared to that before optimization.

Having established the optimal fermentation conditions, the subsequent phase of the research focused on elucidating the antimicrobial mechanism of the Tie-10 crude extract. This study then transitioned to examining the crude extract’s effects on the cell structure of *K. pneumoniae*. Peptidoglycan is the fundamental structural polymer of bacterial cell walls, serving as an indispensable scaffold for cellular integrity and a prime target for clinically important antibiotics [30,31]. This continuous macromolecular network consists of repeating N-acetylmuramic acid and N-acetylglucosamine units crosslinked by short peptide bridges, forming an exoskeleton that counteracts intracellular osmotic pressure [32]. AKP, a periplasmic enzyme localized between the inner membrane and peptidoglycan layer, typically exhibits minimal extracellular release under standard cultivation conditions. Our experimental data demonstrated both temporal and dose-dependent increases in extracellular AKP activity following treatment with Tie-10 crude extract, consistent with prior reports of cell wall-targeting antimicrobials. These findings, corroborated by scanning electron microscopy revealing distinct morphological alterations in *K. pneumoniae* cells, collectively indicated that the antimicrobial mechanism involved substantial disruption of cellular envelope integrity.

In addition to assessing direct physical damage, this study also examined the effects of the crude extract on quorum sensing (QS) in *K. pneumoniae*. QS is a cell-to-cell communication mechanism employed by bacteria to coordinate collective behaviors in response to population density. Through QS, bacteria regulate diverse biological processes, including the secretion of virulence factors, biofilm formation, and the development of resistance to antimicrobial agents [33]. This study systematically investigated the effects of Tie-10 crude extract on *K. pneumoniae* QS through quantitative analysis of extracellular protease activity, comprehensive biofilm formation assays, and qPCR-based profiling of quorum-sensing-related gene expression. Extracellular protease serves as a critical virulence factor in *K. pneumoniae*. This study demonstrated that the crude extract of Tie-10 could attenuate the pathogenicity of *K. pneumoniae* by suppressing its extracellular protease secretion. Biofilms consist largely of extracellular polymeric substances (EPS) secreted by the microbial community, forming a protective matrix that immobilizes bacteria on surfaces and defends them against external threats. These resilient biofilms adhere persistently to various substrates, whether biological or abiotic, creating a fortified microenvironment that promotes microbial survival under harsh conditions. This shielding effect encompasses not only environmental stresses but also enhances tolerance to antimicrobial agents, including antibiotics and antifungals, as well as evasion of host immune defenses during infection [34,35]. Notably, biofilm-embedded microorganisms demonstrated significantly higher resistance than their free-living counterparts. This study demonstrated that the crude extract of Tie-10 exhibits significant inhibitory effects on *K. pneumoniae* biofilm formation. At a concentration of 2 MIC, the extract achieved its maximal biofilm-suppressing activity against *K. pneumoniae*.

A key finding was that the crude extract effectively reduced the expression of several virulent genes in *K. pneumoniae*. Previous studies have demonstrated that deletion of *luxS* in *K. pneumoniae* resulted in structurally compromised biofilms in mutant strains [36]. *wabG* encoded glucosyltransferase, an enzyme essential for lipopolysaccharide (LPS) synthesis, thereby maintaining cellular integrity in *K. pneumoniae*. Furthermore, *wabG* has been shown to disrupt LPS production and impair capsular structure formation [37]. The *fimH* gene encoded a critical virulence factor that mediates host cell adhesion and invasion in *K. pneumoniae*. In this study, we observed that treatment with Tie-10 crude extract significantly downregulated the expression of *luxS*, *wabG*, and *fimH* genes in *K. pneumoniae*, reducing their expression levels to 0.6-fold, 0.19-fold, and 0.1-fold of the control group, respectively.

This study isolated an Antarctic-derived microorganism exhibiting potent antibacterial activity against *K. pneumoniae*, optimized its culture medium and fermentation conditions, and investigated the antimicrobial mechanisms of its crude extract, including effects on the *K. pneumoniae* QS system. These findings provided new perspectives for developing anti-*K. pneumoniae* therapeutics.

## 5. Conclusions

In this study, we isolated a bacterial strain from Antarctic samples demonstrating potent antibacterial activity against *K. pneumoniae*, which was identified as *B. nakamurai* Tie-10 and exhibited broad-spectrum antimicrobial properties. Through culture optimization, we determined that a medium containing beef extract as the carbon source, peptone as the nitrogen source, and potassium nitrate as the inorganic salt yielded the most significant growth inhibition of *K. pneumoniae*. Fermentation parameter optimization revealed that antibacterial activity was most influenced by pH, followed by bottling volume, temperature, and time. The optimal fermentation conditions were established as 24 h incubation at 37 °C, pH 7.0, with a bottling volume of 80 mL. Under these optimized conditions, the inhibition zone diameter reached 28.11 ± 0.54 mm, representing a 2.89 mm increase compared to baseline conditions. Mechanistic studies demonstrated that the Tie-10 crude extract effectively inhibited *K. pneumoniae* growth by disrupting cellular integrity, as evidenced by the increased AKP content in the supernatant. Furthermore, the extract interfered with the virulence of *K. pneumoniae* by suppressing extracellular protease secretion, inhibiting biofilm formation, and downregulating pathogenic gene expression. These findings may provide a foundation for developing novel anti-*K. pneumoniae* therapeutics, contributing to the growing body of research on Antarctic-derived antimicrobial compounds.

## Figures and Tables

**Table 1:** Pathogens used for inhibition spectrum research.

Pathogen	Type of Culture Medium
*Klebsiella pneumoniae*	Nutrient Broth
*Escherichia coli*	LB Broth
*Salmonella typhimurium*	LB Broth
*Staphylococcus aureus*	LB Broth
*Vibrio parahaemolyticu*	LB Broth
*Pseudomonas aeruginosa*	LB Broth
*Acinetobacter baumannii*	Brian Heart Infusion
*Enterococcus faecium*	De Man, Rogosa, and Sharpe
ESBL *E. coli*	LB Broth
Methicillin-resistant *S*. *aureus* (MRSA)	LB Broth

**Table 2:** Factors and levels of Box–Behnken tests.

Levels	Factors			
	A Bottling Volume (mL)	B pH	C Temperature (°C)	D Time (h)
−1	60	6	32	12
0	80	7	37	24
1	100	8	42	36

**Table 3:** qRT-PCR primer sequences.

Primer Name	Primer Sequence (5′~3′)	Primer Size/Bp
*luxS*-F	AGTGATGCCGGAACGCGG	148
*luxS*-R	CGGCGTACCAATCAGGCTC	
*wabG*-F	CGGACTGGCAGATCCATATC	683
*wabG*-R	ACCATCGGCCATTTGATAGA	
*fimH*-F	GCTCTGGCCGATACCACCACGG	423
*fimH*-R	GCGAAGTAACGTGCCTGGAACGG	

**Table 4:** Result of secondary screening.

Strain Number	Inhibition Zone Diameter (mm)	Strain Number	Inhibition Zone Diameter (mm)
TJ-3	21.70 ± 0.29	Tie-36	21.92 ± 0.42
TJ-12	23.47 ± 0.30	qe-15	18.75 ± 0.43
TJ-31	23.78 ± 0.17	qe-35	23.09 ± 0.91
TJ-40	21.61 ± 0.59	qe-45	23.64 ± 0.29
TJ-62	23.19 ± 0.27	C-7	21.96 ± 0.64
Tie-10	25.22 ± 0.27	HB-12	23.36 ± 0.39

Data were presented as the mean ± standard error (SE) of three replicate samples (*n* = 3).

**Table 5:** Antibacterial spectrum of Tie-10.

Pathogen	Inhibition Zone Diameter (mm)	Pathogen	Inhibition Zone Diameter (mm)
*Klebsiella pneumoniae*	25.41 ± 0.44	*Pseudomonas aeruginosa*	16.92 ± 1.09
*Escherichia coli*	25.25 ± 0.12	*Acinetobacter baumannii*	16.63 ± 0.54
*Salmonella typhimurium*	22.95 ± 0.37	*Enterococcus faecium*	15.73 ± 0.77
*Staphylococcus aureus*	17.12 ± 0.95	ESBL *E.coli*	22.11 ± 0.66
*Vibrio parahaemolyticu*	16.97 ± 0.67	MRSA	16.22 ± 0.14

Data were presented as the mean ± standard error (SE) of three replicate samples (*n* = 3).

**Table 6:** Inhibition of *K. pneumoniae* by the crude extract of different concentrations.

Concentration of Crude Extract (mg/mL)	OD_600_	Concentration of Crude Extract (mg/mL)	OD_600_
3.125	0.097 ± 0.003	0.049	0.348 ± 0.059
1.56	0.100 ± 0.005	0.024	0.385 ± 0.064
0.78	0.118 ± 0.009	0.012	0.499 ± 0.036
0.39	0.149 ± 0.016	Negative control	0.047 ± 0.003
0.195	0.188 ± 0.029	Positive control	0.626 ± 0.011
0.098	0.262 ± 0.077	-	-

Data were presented as the mean ± standard error (SE) of three replicate samples (*n* = 3).

**Table 7:** Sample inhibited biofilm of *K. pneumoniae*.

Concentration of Crude Extract	OD_590_	Inhibition Rate (%)
0 MIC	1.348 ± 0.035	-
1/2 MIC	1.225 ± 0.048	9.12%
1 MIC	0.840 ± 0.043	37.69%
2 MIC	0.652 ± 0.020	51.63%

Data were presented as the mean ± standard error (SE) of three replicate samples (*n* = 3).

## Data Availability

The data analyzed in this study are included within the paper.

## Supplementary Materials

The following supporting information can be downloaded at https://www.mdpi.com/article/10.3390/microorganisms13092027/s1. Table S1: Experimental design and results of strain culture conditions; Table S2: Regression analysis of experimental results based on the Box- Behnken design.
