# Supplementary material for: Optimization of Fermentation Conditions for Antarctic Bacteria and Investigation of Its Antimicrobial Mechanism Against Klebsiella pneumoniae
Source: Microorganisms. 2025 Aug 30;13(9):2027. doi: 10.3390/microorganisms13092027 (PMC12472101; doi:10.3390/microorganisms13092027)
Supplement: Supplementary file 1 [file microorganisms-13-02027-s001.zip › microorganisms-3757567-supplementary.pdf]

Optimization of Fermentation Conditions for Antarctic Bacteria and Investigation of Its Antimicrobial Mechanism Against *Klebsiella pneumoniae*

Lukai Xu †, Mengyu Li †, Yangzhu Huang, Yuanchao Mao, Shouyuan Cai, Xinyuan Yang, Xiyan Hou, Lulu Wang, Chunshan Quan * and Liming Jin *

Key Laboratory of Biotechnology and Bioresources Utilization of Ministry of Education, College of Life, Sciences, Dalian Minzu University, Dalian 116600, China; xvlukai@163.com (L.X.); 15825315619@163.com (M.L.); h534198987@163.com (Y.H.); maoyc2023@126.com (Y.M.); 18265502153@163.com (S.C.); syyy-@outlook.com (X.Y.); xyhous@dlnu.edu.cn (X.H.); wanglulu0813@126.com (L.W.)

* Correspondence: mikyeken@dlnu.edu.cn (C.Q.); jlm@dlnu.edu.cn (L.J.); Tel.: +86-186-0986-4172 (C.Q.); +86-135-0408-8982 (L.J.)

† These authors contributed equally to this work.

Table S1 Experimental design and results of strain culture conditions

NO.	A bottling volume(mL)	B pH	C temperature (℃)	D time (h)	Inhibition Zone Diameter (mm)
1	0	0	-1	1	25.85
2	1	-1	0	0	25.77
3	-1	0	1	0	24.11
4	0	0	0	0	28.5
5	-1	0	0	1	22.05
6	1	0	-1	0	25.07
7	0	-1	0	1	25.57
8	0	-1	-1	0	25.78
9	0	0	1	-1	24.05
10	-1	-1	0	0	25.87
11	0	1	0	-1	23.63
12	0	-1	0	-1	24.12
13	0	1	0	1	23.55
14	0	0	0	0	28.11

15	0	-1	1	0	24.88
16	1	0	1	0	26.11
17	0	0	0	0	27.86
18	-1	0	0	-1	24.14
19	1	0	0	-1	23.7
20	0	1	-1	0	24.07
21	-1	0	-1	0	25.36
22	0	0	0	0	27.45
23	0	0	-1	-1	24.31
24	0	0	0	0	27.76
25	1	0	0	1	25.19
26	0	0	1	1	23.48
27	0	1	1	0	24.83
28	1	1	0	0	25.66
29	-1	1	0	0	24.84

Table S2 Regression analysis of experimental results based on the Box- Behnken design

Source	Sum of squares	df	Mean Square	F-value	p-value	Significance
Model	63.75	14	4.55	13.91	<0.0001	**
A	2.19	1	2.19	6.70	0.021	
B	2.44	1	2.44	7.45	0.016	**
C	0.74	1	0.74	2.26	0.155	
D	0.25	1	0.25	0.77	0.394	
AB	0.21	1	0.21	0.65	0.434	
AC	1.31	1	1.31	4.00	0.065	
AD	3.2	1	3.2	9.79	0.007	
BC	0.69	1	0.69	2.10	0.169	
BD	0.59	1	0.59	1.79	0.202	
CD	1.11	1	1.11	3.40	0.086	
A^2	12.72	1	12.72	38.85	<0.0001	
B^2	11.18	1	11.18	34.15	<0.0001	**
C^2	12.65	1	12.65	38.65	<0.0001	**
D^2	38.28	1	38.28	116.9	<0.0001	**
Residual	4.58	14	0.3274			
Lock of Fit	3.96	10	0.3963	2.55	0.190	

Pure of Error	0.6213	4	0.1553
Cor Total	68.33	28	
		R^2=0.9329	R^2_{adj}=0.8658
